# Editorial: A global perspective on diversity in eating disorders

**DOI:** 10.3389/fpsyt.2023.1276078

**Published:** 2023-08-29

**Authors:** Georgios Paslakis, Gina Dimitropoulos, Georg Halbeisen

**Affiliations:** ^1^University Clinic for Psychosomatic Medicine and Psychotherapy, Medical Faculty, Campus East-Westphalia, Ruhr-University Bochum, Lübbecke, Germany; ^2^Faculty of Social Work, University of Calgary, Calgary, AB, Canada

**Keywords:** anorexia nervosa, bulimia nervosa, binge-eating disorder, social diversity, gender bias, ethnic bias

## 1. Introduction

Eating disorders (EDs) are characterized by body image disturbances, abnormal eating, and weight-control behaviors associated with adverse physical and mental health outcomes across multiple domains of functioning (1, 2). EDs can pose one of the highest mortality risks among psychiatric disorders (3), can often be difficult to treat (4), and incur substantial healthcare and socio-economic costs (5, 6). Counter to the widespread perception that EDs affect mostly Caucasian, adolescent girls from wealthy and Western industrialized countries, recent years witnessed an unprecedented increase in ED incidence among individuals of diverse genders, ethnicities, and socio-economic backgrounds (7). About 1.7% of the global population suffers from an ED during their lifetime, with the current COVID-19 pandemic and public health restrictions likely resulting in further spikes across the globe (8). Identifying differences in risk factors and ED presentation and developing diversity-affirming treatments is therefore urgently needed. This Research Topic brings together scholars from across the globe to share and combine their research perspectives on diversity in eating disorders.

Thus far, differences in ED presentation between Western and non-Western countries remain barely explored. Advancing our knowledge in this domain, An et al. studied biochemical, hematologic, and skeletal features cross-sectionally in more than 800 young women in South Korea, including women with extreme weights and EDs (anorexia nervosa and bulimia nervosa). Their findings suggest that overall, physiological parameters in Korean patients with EDs were similar to the patterns reported in Western samples in previous studies, with few exceptions such as higher potassium levels in patients with bulimia nervosa. These could be attributed to the binging of potassium-rich Korean foods, which hints at a potentially relevant role of cultural differences in environmental factors that contribute to ED etiology and presentation.

Investigating the role of environmental factors further, Hemmingsen et al. explored associations between re-nutrition, elevated cortisol levels, and depression and anxiety symptomatology in patients with anorexia nervosa. The authors hypothesized that cortisol levels and depression and anxiety symptomatology would decrease following intensive re-nutrition, but findings from a prospective observational study with patients in Denmark could not confirm these expectations. It might be that short-term intensive re-nutrition is not sufficient to have a measurable effect on cortisol levels and psychometrics in patients with severe anorexia nervosa. The study highlights further avenues for exploring the triggering factors of eating disorders onset, and extending the exploration of cultural differences in eating disorders treatment.

Inspired by cross-cultural disparities in treatment effectiveness for patients with bulimia nervosa, Hamatani et al. took up the challenge of systematically adapting an English internet-based cognitive behavioral therapy program for patients in Japan. The researchers first describe the initial process of translating and adapting linguistic expressions, concepts, metaphors, and dietary contents for patients in Japan, which highlights that even minor details, such as giving positive feedback, may require critical review for cultural appropriateness. The researchers then gathered evaluations by patients and clinicians, and used this feedback to further improve upon the cultural appropriateness of the therapy program. This three-step program could serve as a blue print for future cultural adaptations of ED treatment, and further highlights the need to improve diversity-related infrastructure and content in treatment settings.

Expanding upon cultural differences, Traut et al. explored diversity in terms of gender differences in EDs. The researchers compared 104 men to 104 diagnosis-matched women with EDs from a German eating disorders clinic regarding sociodemographic and clinical features. Using latent class mixture modeling, four distinct patient subgroups emerged, with men with EDs having a significantly higher odds than women to belong to a “single-childfree-working” class. Moreover, while there were few overall differences in ED-related symptoms and general psychopathology between men and women, single-childfree-working men with EDs presented with higher general psychopathology symptoms than men in the other classes. The study hints at a critical role of intersecting diversity perspectives (gender and socio-economic status), which thus far remain barely addressed in research and care.

There have been numerous advances toward understanding the role of diversity in ED etiology and access to care in recent years (7). Complementing these efforts, this Research Topic presents recent findings on cultural and gender differences in ED presentation and treatment. The studies illustrate how environmental factors may affect the clinical presentation of patients with EDs, how cultural sensitivities may shape treatment approaches and their effectiveness, and how different facets of diversity may interact to amplify symptom burden. Taken together, the Research Topic stipulates future research avenues, which could ultimately improve the detection and care of individuals with EDs worldwide.

## Author contributions

GP: Writing—original draft, Writing—review and editing. GD: Writing—original draft, Writing—review and editing. GH: Writing—original draft, Writing—review and editing.
